# Editorial: Cellular Stress and Inflammation: How the Immune System Drives Tissue Homeostasis

**DOI:** 10.3389/fimmu.2021.668876

**Published:** 2021-03-18

**Authors:** Fabrizio Antonangeli, Ola Grimsholm, Marianna Nicoletta Rossi, Francesca Velotti

**Affiliations:** ^1^ Department of Molecular Medicine, Sapienza University of Rome, Laboratory Affiliated to Istituto Pasteur Italia - Fondazione Cenci Bolognetti, Rome, Italy; ^2^ Institute of Molecular Biology and Pathology, National Research Council (CNR), Rome, Italy; ^3^ Department of Rheumatology and Inflammation Research, The Sahlgrenska Academy, University of Gothenburg, Gothenburg, Sweden; ^4^ Laboratory of Immuno-Rheumatology, Bambino Gesù Children’s Hospital, IRCCS, Rome, Italy; ^5^ Department of Ecological and Biological Sciences (DEB), Tuscia University, Viterbo, Italy

**Keywords:** inflammation, immune system, tissue homeostasis, metabolism, stress response, fibrosis, PD-L1, granzymes

For a long time, the immune system (IS) has been referred to only as a defense mechanism against pathogens and external threats. Nowadays, its pivotal role in orchestrating tissue remodeling of multicellular organisms is undoubtful. Cells facing cellular stress, by expressing stress-induced molecules or through the release of a plethora of soluble factors, undertake a dialog with the IS. In this way, cells link intracellular stress to systemic homeostasis, a process that has likely evolved during the establishment of the colonial way of life.

In this *Frontiers in Immunology* Research Topic, the interaction of parenchyma cells with the IS during peculiar inflammatory processes has been addressed by leading investigators, providing different point of views on the contribution of the IS to tissue homeostasis. Fibrosis is a prototypical response of damaged tissues largely guided by the IS, here well described by Trionfetti et al. in the peritoneum. The authors highlight the complex network among stromal cells, resident and recirculating leukocytes, which sustain peritoneal fibrosis. Mesothelial cell plasticity is at the base of the fibrotic process and is mostly affected by the cytokine milieu produced by locally activated or recruited immune cells. In particular, the crosstalk between mesothelial cells and macrophages is characterized by a bidirectional mode of action.

Antonangeli et al., considering the evidence that the key inflammatory mediator NF-κB is emerging as a positive regulator of PD-L1 expression in cancer, suggest that PD-L1 upregulation takes part in cancer biology as a maladaptive consequence of its physiological role during inflammation, namely by mirroring a process aimed at restoring tissue integrity during epithelial stress response, alike the epithelial to mesenchymal transition (EMT). It is conceivable that tissue morphogenesis, inflammation and immune response have a common evolutionary root, with NF-κB representing the link and the immune checkpoint PD-L1 a consequence.

The contribution by Mandatori et al. is focused on regulatory T cells (Tregs) during atherogenesis, a pathological process of the blood vessels that largely involves the IS. The authors show that autophagy sustains Treg maturation and they hypothesize that an impairment of the autophagic processes, by affecting Tregs, contributes to the inflammation of the atherosclerotic plaques. The article sheds light to the intricated relationship between metabolism and the IS.

In this collection, two other peculiar aspects of the interplay between cellular metabolism and the IS are highlighted. Bilotta et al. summarize several studies showing how cholesterol and cholesterol metabolism influence immunity and inflammation. Indeed, the cholesterol receptors LXRs are able, depending on the cellular context, to act as activators or repressors of genes regulating different cellular responses including apoptosis, cytokine production, and cell cycle inhibition. These effects are particularly relevant to autoimmune diseases such as systemic lupus erythematosus, rheumatoid arthritis, and atherosclerosis. Moreover, the cholesterol metabolism can also play a role in the regulation of tumor growth, as demonstrated in ovarian, colon, and prostatic cancers.

In the review article by Lauro and Limatola the metabolism-IS interconnection is analyzed in the context of neuroinflammation. Microglia, the sentries of the central nervous system, undergo a metabolic switch depending on the activation status. It is emerging that homeostatic microglia are characterized by an oxidative metabolism, while reactive microglia (similarly to activated macrophages) switch towards a glycolytic and pentose phosphate metabolism to support cell proliferation. This finding is mostly important because reactive microglia release cytokines and free radicals contributing to brain neurodegeneration and inflammation, while homeostatic microglia promote brain repair and has anti-inflammatory properties. Thus, targeting microglia metabolism represents an intriguing opportunity for the treatment of neurodegenerative diseases.

Macrophages are crucial in tissue homeostasis by catching apoptotic cells, removing cellular debris, and secreting cytokines. Two studies in this Research Topic pay attention to alveolar macrophages (AMs). AMs from pulmonary surfactant secretoglobin family 1A member 1 (SCGB1A1) deficient mice show alterations in different inflammatory pathways as evaluated by high-throughput RNA-sequencing and gene expression analysis. Xu et al. suggest that SCGB1A1 is necessary for AMs to stimulate an appropriate adaptive immune response and that SCGB1A1 supplementation could reduce cytokine surge during airway infections.

AMs control neutrophil homeostasis in pulmonary alveoli by expressing neutrophil chemotactic factors and by clearing apoptotic neutrophils (a process called efferocytosis). Alpha 1 antitrypsin deficiency (AATD) is a genetic disorder characterized by neutrophil accumulation in the lungs. Lee et al. propose a mechanism by which the mutated allelic variant of AAT, Z-AAT, is retained intracellularly and induces the expression of CXCL8 and TNF-α by AMs. CXCL8 is responsible for the increased neutrophil recruitment while TNF-α, by affecting CD14, CD36, and RARα, reduces AM efferocytosis ability, thus contributing to the pulmonary disease in AATD individuals.

Innovative considerations come from three articles included in the present collection that are all focused on the multifunctional pro-inflammatory role of granzymes (Gzms), a family of serine proteases expressed by immune, non-immune, and tumor cells. In particular, the involvement of Gzms in the pathogenesis of serious inflammatory diseases highlights the potential therapeutic effect of targeting Gzms to reduce organ damage. Garzòn-Tituaña et al. present evidence suggesting the relevant role of Gzms in sepsis pathophysiology. The authors show the role of GzmA and GzmM in regulating the inflammatory cytokine network, contributing to the cytokine storm, as well as in the regulation of the coagulation cascade, platelet function and endothelial barrier permeability, during the hyper-inflammatory initial-stage sepsis. Furthermore, a possible role of GzmB in the immunosuppressive late-stage sepsis is discussed.

In a research article, Cimini et al. nicely describe the expression of GzmB in both visceral adipose tissue (VAT) and serum of obese patients and report its association with VAT inflammation and glucose homeostasis dysregulation, suggesting that GzmB pro-apoptotic and extracellular proteolytic activities contribute to obesity-related VAT inflammatory remodeling and fibrosis, leading to adipose tissue dysfunction in metabolic diseases.

Finally, Velotti et al. provide an overview on recent data concerning the multifunctional pro-inflammatory activity of GzmB, consisting of perforin-dependent and perforin-independent (anoikis) apoptosis, IL-1 cytokine family activation, extracellular matrix remodeling, induction of EMT and fibrosis, that might underlie inflammaging and the pathogenicity and/or severity of acute and chronic inflammatory diseases, such as cardiovascular, pulmonary, metabolic diseases, and cancer.

Altogether, the articles included in the present Research Topic provide a fascinating view of the IS as a key player of tissue homeostasis, hopefully inspiring new developments in this challenging field. The articles well describe the plasticity of the IS in both physiological and pathological conditions. As a result, the idea of exploiting the plasticity of the IS, by altering cell metabolism and cell polarization or by targeting specific component, is emerging, with the aim of counteracting pathologies characterized by tissue homeostasis dysregulation such as cancer, neurodegenerative, and metabolic diseases.

## Author Contributions

FA, OG, MNR and FV edited the topic and wrote the manuscript. All authors contributed to the article and approved the submitted version.

## Conflict of Interest

The authors declare that the research was conducted in the absence of any commercial or financial relationships that could be construed as a potential conflict of interest.

